# A Treatise on the Nature and Treatment of the Hooping-Cough, and Its Complications, Illustrated by Cases

**Published:** 1839-01

**Authors:** 


### ART. XI.

*A Treatise on the Nature and Treatment of the Hooping-cough, and its Complications, illustrated by Cases: with an Appendix, containing Hints on the Management of Children.* By G. H. ROE, M.D. Oxon, Physician to the Westminster Hospital.—*London*, 1838. 8vo. pp. 258.

THE variety of plans of treatment and of remedies, avowed and secret, for hooping-cough, evinces either that this disease runs its course uncontrolled by art, or that its pathology is still unsettled. We believe the latter to be the case, and are therefore the more disposed to pay attention to the present work, though possessing no claims to originality, because it furnishes us with an opportunity of calling the notice of our readers to the present position of the question, and of showing that a field of investigation is still open to other pathologists.

The prevalent opinions respecting the essential nature of hooping-cough admit of division into two great classes,—according to one of which it is a neurosis, according to the other an inflammation: the former class rest on the phenomena observed during life, the latter on the organic changes discovered after death. It is obvious that those who regard this affection as nervous will generally be found to precede, in point of time, those who maintain the inflammatory doctrine; the disposition to scrutinize our organs for the causes of disease being of late years much increased. Of the advocates of the former opinion one of the most celebrated is Hufeland, who, in 1793, influenced probably by the convulsive nature of the symptoms, attributed hooping-cough to an irritation of the eighth pair and the phrenic nerves: he maintained that, when this irritation was extreme, it extended to the cardia, and vomiting ensues, which removes the irritation from the pulmonary nerves, and the paroxysm ceases. It was never understood that these opinions of Hufeland underwent any modification from the diffusion of cadaveric researches or other circumstances; and, had they been modified, the public, from the learned professor's constant connexion with the medical press of Germany, would certainly have been acquainted with the change. It must be manifest that a doctrine sanctioned by such high authority would obtain an influence quite independent of its intrinsic reasonableness; and it will not be matter of surprise that Jahn, Löbenstein-Loebel, and the majority of German practitioners, advocated the nervous theory of the disease. A similar theory prevailed in this country from the days of Cullen till a comparatively recent period, when pathological anatomy came to be generally considered as the means by which the intricacies of the disease were to be unravelled.

But, besides the light derived from this source, there were circumstances attending the disease, which, not receiving an explanation from a nervous pathology, led many persons to consider it as partaking of the nature of inflammation. The symptoms of catarrh, an inflammatory disease, precede for some time the characteristic cough; and, both during this period and afterwards, in the interval of the fits of convulsive coughing, the mucous wheeze, and occasionally the other rhonchi which distinguish pulmonary catarrh, are perceptible. Hence there was an impression, either that such catarrh or the secretion it occasioned gave rise to the convulsive cough; and the latter of these views was considerably confirmed by the relief afforded by the expulsion of mucus during the spontaneous vomiting common in the disease, or that artificially produced. The main ground, however, for the substitution of an inflammatory for a nervous doctrine were the visible marks of an inflammatory condition displayed on cadaveric inspection; and to Dr. Watt, of Glasgow, is due the credit of having been the first in this country to draw the attention of the profession to such marks in the air-passages and lungs in this disease.

The circumstance of ordinary bronchitis and pneumonia being unaccompanied by the peculiar cough of pertussis, constitutes an obvious objection to the doctrine which would identify it with these affections; and some writers, struck with the discrepancy, and moreover impressed by the manifest cerebral affection existing in a proportion of cases, have placed the site of the inflammation, irritation, or congestion, supposed to characterize the disease, in the brain. This opinion was supported by Dr. Webster in our own country, and by M. Alphonse Leroy in France; and, in 1827, M. Desruelles advocated the same doctrine, with considerable force of argument, in his "Traité de la Coqueluche." "So long," says he, "as the bronchitis is simple, the cough presents no peculiarity; but when the diaphragm, the muscles of respiration, those of the glottis, of the larynx, the posterior membrane of the bronchi, the air-cells of the lungs, and even, according to Laennec, those of the *velum palati*, come into action, and are simultaneously affected with spasm, under the influence of the *cerebral irritation*, the cough changes its character, and becomes convulsive; and every time that an afflux of blood takes place to the brain the cough returns, and appears in paroxysms. This intermittent congestion precedes the kink of coughing, and disappears along with it, to reappear shortly, and to bring on a fresh paroxysm."*

After giving a candid exposition and examination of these various doctrines, Dr. Roe states his own opinion in the following terms:

"All we know is, that a spasmodic affection of the muscles in question, and a slight inflammation of the air-passages, are the first effects produced by the cause of hooping-cough, when that disease exists without complication; and that the violence with which the blood is driven throughout the body frequently causes inflammation of some other parts, and by degrees brings on that fearful train of consequences which we have seen to be so often the result of the long continuance of the cough." (p. 67.)

Dr. Roe is struck with the coincidence between his opinion and that expressed by M. Blache, in the Archives générales de Médecine for 1833, who considers hooping-cough as a nervous affection very frequently complicated with bronchitis or pneumonia, but which may exist without them. There is this striking difference between them, that the opinion of Dr. Roe involves the hypothesis that all inflammatory affections existing in this disease, slight bronchitis excepted, are produced by the violence with which the blood is propelled through the body by the cough; whilst M. Blache more prudently confines himself to expressing the fact of the coincidence of the nervous affection and inflammation. It is not only not proved, but it is in the highest degree improbable, that the more intense degrees of bronchitis, and the pneumonia still more frequently attending the disease, are produced by the cough; though we think that the cough and the affection of respiration during the paroxysms, furnish a reasonable explanation of the cerebral affection when such occurs, and one, too, supported by some analogies; such, for instance, as that of influenza when it attacks infants.

After his very lengthened disquisition on the cause and seat of the disease, Dr. Roe passes to the consideration of the more practical part of his work. He first considers simple hooping-cough, and then its complications with bronchitis, pneumonia, hydrocephalus, and remittent fever; all these divisions displaying but too clearly that dearth of original observation and reasoning which characterizes the volume. One of

* Traité, p. 77.

his patients, labouring under what was considered simple hooping-cough, dies somewhat unexpectedly,—we think it probable from interlobular pneumonia. Permission is granted to examine the body, but, from regard to the feelings of parents, Dr. Roe declines doing it. Now, we think a kind of humanity much superior to that so indulged would have suggested an examination. It is likewise to be regretted that, amid such poverty of original observation, the smallest particle should have been omitted. We would ask why pleuritis is omitted from the list of complications? It is one which has more than once fallen under our own observation in dissection, and has tended to confirm the view we have long entertained, that there is in this disease an inherent (not an accidental) tendency to inflammation of the thoracic viscera.

The author's opinions on the treatment of the disease are developed principally in the eighth chapter, or that on Simple Hooping-cough. His favorite remedy is hydrocyanic acid; the adoption of which he owes to a work on this medicine, published by Dr. Granville a good many years ago. Dr. Roe combines it with ipecacuanha or tartarized antimony, but ascribes to the acid alone the power of curing the disease. He informs us that, two or three days after the employment of these medicines, the violence of the paroxysms is perceptibly diminished, and their duration is shortened; and at the end of five or six days the whoop ceases. This section of the work shows Dr. Roe to be a judicious physician; and, had it been published in some journal, would have done him credit. His error has consisted in swelling the materials of an *article* into a volume of nearly three hundred pages, by gleanings from works with which the public was already well acquainted. The evidence adduced of the utility of the medicine is very decisive, and the rules for its employment are generally judicious. Perhaps the following statement must be regarded as an exception to this claim to praise; the more so, as it appears from some passages in the book that non-medical persons have given the medicine to their children on the authority of Dr. Roe:

"The dose should be repeated when the effects begin to subside, which, in mild cases, generally happens in three or four hours; but, when much fever is present, its influence is felt but a very short time: under such circumstances a larger quantity may be given, and at shorter intervals, without any apprehension of danger, *so long as the fever lasts. In some very severe cases,* when the pulse was up to 120, with a good deal of fever and a very hot skin, I have given to *a girl of ten years of age* a minim and a half of this medicine every quarter of an hour, for twelve hours: at the end of twenty-four hours she was free from fever, and her strength was not in the least reduced by the effects of the remedy." (p. 90.)

We have witnessed much of the tolerance of powerful medicines by persons in a state of febrile excitement; but we were not aware that it had ever been attempted, under any circumstances, to give a child of ten years of age seventy-two minims of hydrocyanic acid in twelve hours. We hope that a skilful and vigilant medical man sits by to superintend the administration of these powerful doses, and that no clerical or matronly reader of Dr. Roe's work will imitate them.

We feel ourselves compelled to pass over the ninth, tenth, and eleventh chapters, which treat of the complication of hooping-cough with bronchitis, pneumonia, and hydrocephalus, with the comment that "all is barren" of interesting and instructive matter. The twelfth chapter, treating of "general rules for the management of hooping-cough," displays that common-place judiciousness which constitutes the useful physician, but not a writer of books and instructor of his fellows. The same dull mediocrity pervades the concluding fifty pages of the volume, which are dedicated to hints on the general management of children. In the work of a member of our most learned university one naturally looks for graces of style to adorn what is accurate, and relieve what is faulty in reasoning; but here, too, we regret to say that we are doomed to disappointment. The composition is certainly far from elegant, and is not always even accurate.

---

